# Diabetes Insipidus in Mice with a Mutation in Aquaporin-2 

**DOI:** 10.1371/journal.pgen.0010020

**Published:** 2005-08-19

**Authors:** David J Lloyd, Frank Wesley Hall, Lisa M Tarantino, Nicholas Gekakis

**Affiliations:** 1 Genomics Institute of the Novartis Research Foundation, La Jolla, California, United States of America; 2 Department of Pathology, Scripps Clinic, La Jolla, California, United States of America; Harvard Medical School, United States of America

## Abstract

Congenital nephrogenic diabetes insipidus (NDI) is a disease characterized by failure of the kidney to concentrate urine in response to vasopressin. Human kindreds with nephrogenic diabetes insipidus have been found to harbor mutations in the *vasopressin receptor 2 (Avpr2)* gene or the vasopressin-sensitive water channel *aquaporin-2 (Aqp2)* gene. Development of a treatment is rendered difficult due to the lack of a viable animal model. Through forward genetic screening of ethylnitrosourea-mutagenized mice, we report the identification and characterization of a mouse model of NDI, with an F204V mutation in the *Aqp2* gene. Unlike previously attempted murine models of NDI, our mice survive to adulthood and more exactly recapitulate the human disorder. Previous in vitro experiments using renal cell lines suggest recessive *Aqp2* mutations result in improper trafficking of the mutant water pore. Using these animals, we have directly proven this hypothesis of improper AQP2 translocation as the molecular defect in nephrogenic diabetes insipidus in the intact organism. Additionally, using a renal cell line we show that the mutated protein, AQP2-F204V, is retained in the endoplasmic reticulum and that this abnormal localization can be rescued by wild-type protein. This novel mouse model allows for further mechanistic studies as well as testing of pharmacological and gene therapies for NDI.

## Synopsis

Nephrogenic diabetes insipidus (NDI) is a disease marked by excessive urination and thirst. Normally, the hypothalamus senses situations where water is limited and signals to the kidney to increase water reabsorption from urine. The signaling molecule secreted by the hypothalamus is arginine vasopressin (AVP), which binds to a specific protein on the surface of kidney cells, AVP receptor (AVPR2). AVP binding to its receptor on kidney cells begins a series of biochemical events that ultimately results in the insertion of a protein, aquaporin 2 (AQP2), into the outer surface of the kidney cell. As its name suggests, AQP2 facilitates the reuptake of water from the urinary space into the cell, thus concentrating the urine and conserving water. Congenital NDI is caused by mutations in either the water channel, AQP2, or in the receptor, AVPR2. While these mutations have been studied extensively in the lab, work in live animals has been very limited. This report describes the first viable mouse model of NDI. Previous models have been attempted by targeted mutation, i.e., genes known to be involved in the disease have been altered in the mouse, a so-called reverse genetic approach. Reverse genetic approaches have so far failed to produce a viable mouse model of NDI. Here the authors take a forward genetic approach in which genes are mutated at random and animals are screened for disease-like properties. As well as proving hypotheses that come from lab studies, this model opens the door to the testing of gene therapy or other therapies for treatment of NDI.

## Introduction

Nephrogenic diabetes insipidus (NDI) is a disease characterized by excessive urination and thirst, despite normal production of the antidiuretic hormone arginine vasopressin (AVP) [1]. The inherited forms are either X-linked as a consequence of mutation of the *Avpr2* gene [2], or autosomal due to mutation of the *Aqp2* gene [3]. Aquaporin-2 (AQP2) is a pore-forming protein belonging to a family of water channels [4], and it is expressed in collecting-duct principal cells in the kidney [5]. Generally these proteins permit the passage of water through the plasma membrane (PM) of cells, several of which carry out this role specifically in the process of water reabsorption from urine in the kidney. It has been established that aquaporins, although functional as a monomer, tetramerize before their insertion into the plasma membrane [4,6]. Furthermore these proteins can also be differentially targeted to distinct regions of the PM; for example, AQP2 is routed to the apical membrane of cells surrounding the collecting duct, whereas other aquaporins (AQP3 or 4) are inserted into the basolateral face. Unlike all other family members, AQP2 is not constitutively inserted into the plasma membrane. Under basal conditions, the protein resides in subapical intracellular vesicles; however, under conditions requiring water retention AQP2 translocates to the apical membrane, permitting water reabsorption [7,8]. For this process to occur, AVP binds its receptor, AVPR2, on the basolateral face of the collecting duct cells, leading to a rise in intracellular cAMP, ultimately resulting in phosphorylation of AQP2 at serine 256 by cAMP-dependent protein kinase [9] and its redistribution to the plasma membrane.

The importance of AQP2 redistribution has been highlighted by functional characterization of *Aqp2* mutations resulting in severe NDI in humans [3,10]. Recessive *Aqp2* mutations are generally thought to produce an abnormally localized and, in most instances, misfolded water pore that responds abnormally to an increase in cAMP [6,11]. Furthermore, dominant mutations have been described and found to misroute both the mutant and the wild-type protein to the basolateral membrane [6,12].

Several mouse models of diabetes insipidus have been generated [13–17]. In an attempt to recapitulate human NDI, mice have been generated with mutations in *Aqp2* and *Avpr2* [15,18]. Yang and colleagues created a mouse with a T126M knock-in mutation in the *Aqp2* gene. Unexpectedly, homozygous mutant mice died within 6 d after birth. Interestingly, AVPR2-deficient male pups also die within the first week after birth. Together these models suggest that the mouse may be a highly sensitive organism with regard to water homeostasis, and is unable to survive with polyuria.

In a forward genetic screen, a mouse with an *Aqp2* mutation was identified. The purpose of this study was to characterize this murine model of recessive nephrogenic DI. We now report a novel F204V mutation in the *Aqp2* gene. This allele of *Aqp2* was found to cause the first mouse model of NDI to survive past the first week of life. Molecular analyses concluded that mutant AQP2 adopts a different subcellular localization in renal collecting-duct cells, and was resistant to translocation induced by desmopressin, an agonist of AVP. In vitro studies using the Madin-Darby canine kidney (MDCK) cell line demonstrated an endoplasmic reticulum pattern for the mutant protein, and apparent resistance to translocation. These data conclusively prove that autosomal recessive NDI is a consequence of improper AQP2 routing in the intact mammal.

## Results

In a forward genetic screen that used ethylnitrosourea (ENU) to induce mutations in a founder animal whose offspring were then screened for abnormal whole body metabolism [19,20], we found a family of mice that urinated and drank excessively. Serum and urine analysis showed that plasma glucose levels were normal and there was no glucose in the urine (unpublished data). Hence, this was an example of diabetes insipidus.

The disorder in these mice segregated in a monogenic, autosomal recessive manner, making *Aqp2* a candidate gene. Sequencing of *Aqp2* coding region of affected mice identified a thymine to guanine (T to G) transversion (Figure 1A), which is predicted to lead to a valine for phenylalanine substitution at amino acid 204 of the protein (F204V).

**Figure 1:**
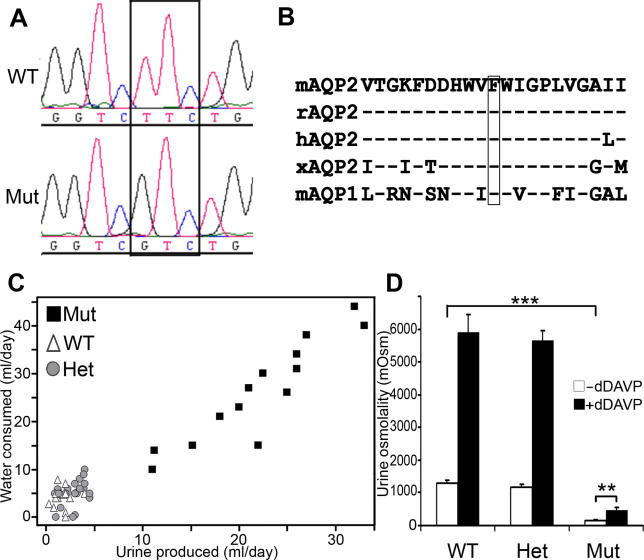
Analysis of *Aqp2* Sequence and Phenotype in Mutant Mice (A) Chromatographic traces of *Aqp2* F204V mutation. The box shows the mutated codon, TTC (Phe) to GTC (Val) at position 204. WT, wild type; Mut, mutant. (B) Amino acid conservation of mouse AQP2 (residues 194–214). The boxed residue indicates phenylalanine at position 204. hAQP2, human AQP2; mAQP1, mouse AQP1; mAQP2, mouse AQP2; rAQP2, rat AQP2; xAQP2, *Xenopus* AQP2. (C) Urine production (ml) and water consumption (ml) of 58 F2 mice over a 24-h period (both sexes, aged 10–22 wk). Mutant mice (black squares) exhibit overt polyuria and polydipsia compared to littermate wild-type (white triangles) and heterozygous (grey circles) mice. (D) Urine osmolality and concentrating ability in *Aqp2* mutant and their littermates (10–22 wk, both sexes), before (white bars) and after (black bars) dDAVP treatment. Wild type (WT; *n* = 12); heterozygote (Het; *n* = 20); mutant (Mut; *n* = 9). Data represent averages ± standard error of the mean, **p < 0.01; ***p < 0.001.

AQP2 is a six-transmembrane water channel, and F204 lies near the extracellular face of the sixth membrane spanning domain, a region rich in hydrophobic amino acids. This and the other membrane-spanning domains are conserved among vertebrate species. The phenylalanine at position 204 is particularly well conserved (Figure 1B), not only among vertebrate AQP2 proteins, but also among others members of this family.

*Aqp2^F204V/F204V^* mice have dramatically increased urine production, in some cases producing an amount of urine in 24 h that exceeds their body weight, compared to their heterozygous or wild-type littermates. Such loss of water would rapidly lead to dehydration were it not compensated by increased water intake. Indeed, mutant mice also dramatically increase their water intake (Figure 1C) compared to their heterozygous or wild-type littermates. This phenotype—increased urinary output and water intake—showed complete concordance with homozygosity of the F204V mutation in the 58 animals tested.

Diabetes insipidus can be defined as an inability to concentrate urine where appropriate. Compared to wild-type or heterozygous littermates, *Aqp2^F204V/F204V^* mice produce very dilute urine (Figure 1D). Basal urine concentration in mutant mice is about 161 mOsm, compared to about 1,293 mOsm in wild-type mice (*p* < 0.001). Normally, urine concentration is under the control of the hypothalamus, which, in response to hypovolemia or hypernatremia [21], secretes AVP. The synthetic AVP analog, 1-deamino-8-D-arginine vasopressin (dDAVP; also called desmopressin), is a potent agonist of AVPR2. When administered to wild-type mice, dDAVP leads to a dramatic increase in urine concentration, from 1,293 to 5,885 mOsm (4.6-fold; Figure 1D). With similar treatment, mutant mice concentrate their urine to a lesser but still significant extent, from 161 to 470 mOsm (2.9-fold), indicating that these animals are not only unable to concentrate their urine properly but are also defective in their response to dDAVP. The smaller response to dDAVP indicates some residual activity of the mutant AQP2 channel, which must be sufficient to allow survival of the individual, in contrast to the T126M knock-in mouse [18].

Multiple heterozygous matings yielded 101 animals, which appeared at a ratio of 26:49:26, near the expected Mendelian wild type, heterozygote, and mutant frequencies, respectively, indicating that there is no reduced viability associated with this mutation. Other than the increased urine production and water intake, there was no overt phenotype in mutant mice, save distended kidneys, which appeared variably in adult animals (Figure 2A). Although not specifically measured, mutant mice seem to have a normal lifespan. The one animal that was followed lived to 18 mo, typical for animals in our colony.

**Figure 2:**
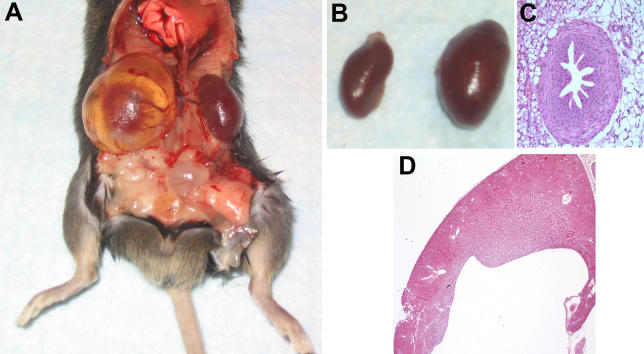
Anatomy and Histology of Mouse Kidneys (A) Gross anatomy of an affected mouse (8-mo-old male). This shows the enlargement and cystic dilatation of the renal pelvis. There is thinning of the overlying renal parenchyma imparting a translucent appearance to portions of the kidney and collecting system. The bladder is also dilated. (B) Left kidney from mutant mouse (right) shown in (A) compared to a kidney from an age-sex matched unaffected littermate (left). (C) Hematoxylin and eosin stained section of ureter from a mutant mouse, showing normal histology despite bloating of the kidney. (D) Hematoxylin and eosin stained histologic section of a kidney from a 4-wk-old female mutant mouse. The mutant kidney shows marked dilatation of the renal pelvis with blunting of the papilla. There is preservation of the cortex and medulla.

*Aqp2^F204V/F204V^* mice suffer from severe hydronephrosis (Figure 2A and 2B), presumably as a consequence of an inability to cope with the extreme polyuria. We found distended kidneys in all *Aqp2^F204V/F204V^* mice; however, the degree of inflation was variable in affected mice and worsened with age. Severe hydronephrosis has previously been observed in double *Aqp1/Aqp3* knock-out mice [17], and appears at 6 wk. Even at 4 wk, *Aqp2^F204V/F204V^* mice had hydronephrosis. Histologic sections from *Aqp2^F204V/F204V^* mice demonstrated marked dilatation of the renal pelvis yet normal morphology of the ureter (Figure 2C and 2D). In particular, the muscularis propria was neither hypertrophied nor thinned. There was the normal festooned appearance of the urothelium, and this transitional epithelium was of normal thickness. There was thinning of the kidney as measured from renal capsule to renal pelvis. However, the morphologic features of the glomeruli and proximal/distal tubules were unremarkable (Figure 2D).

As shown previously [18,22], immunoblotting revealed three different forms of AQP2, due to different degrees and forms of glycosylation (Figure 3A). Previous reports have demonstrated that nonglycosylated protein appears as a 29 kDa band, while complex glycosylated protein runs as a smear between 35 and 45 kDa. A short-lived intermediate form of 31 kDa representing core, high-mannose glycosylation of AQP2 is apparent from pulse-chase labeling experiments [22]. Compared to that from the kidneys of wild-type animals, AQP2 from mutant animals was reduced in both the high molecular weight, diffuse form and the lowest molecular weight form, but enriched in the intermediate molecular weight form (Figure 3A). Heterozygous animals showed intermediate amounts of all three forms. The nature of these glycosylated forms was revealed by digestion with endoglycosidase H, which specifically cleaves mannose-rich carbohydrate from the protein backbone. Treatment of endogenous AQP2 from kidneys of wild-type, heterozygous, and mutant animals specifically affected the intermediate molecular weight form (Figure 3B). The presence of some mature glycosylated proteins (35–45 kDa) in *Aqp2^F204V/F204V^* mice presumably permits their survival compared to *Aqp2^T126M/T126M^* mice, and is consistent with a diminished response to dDAVP.

**Figure 3:**
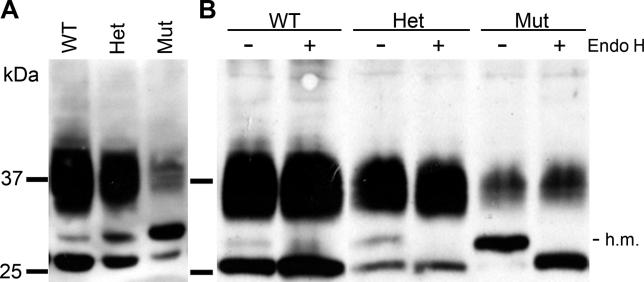
Immunoblot Analyses of AQP2 from Mouse Kidneys (A) Western blot analyses of total kidney membranes from littermate mice. An intermediate form of AQP2 at 31 kDa was identified in kidney membranes from a mutant mouse (Mut) and partially in a heterozygous mouse (Het). (B) Total kidney membranes were subjected to endoglycosidase H treatment (Endo H) prior to Western blotting. High-mannose (h.m.) glycosylated proteins that have not exited the ER are sensitive to endoglycosidase H digestion.

In humans, recessive alleles of *Aqp2* are postulated to cause NDI because they do not properly translocate to the apical cell surface in response to AVP. This postulate comes solely from in vitro studies in which mutant *Aqp2* cDNAs corresponding to human disease mutations are transfected into kidney cell lines. In general, such recessive alleles, when visualized immunocytochemically, fail to localize to AVP-responsive vesicles. Rather, they get trapped in the endoplasmic reticulum (ER). Our mouse model of NDI affords the first opportunity to test this hypothesis in a mature animal. As shown in Figure 4A (top row of photomicrographs), AQP2 (stained red) normally localized to the subapical region of collecting duct cells in kidneys of wild-type mice. Upon stimulation with dDAVP, AQP2 translocated to or near the cell surface (Figure 4A, second row). In kidneys taken from mutant animals, however, AQP2 was distributed randomly throughout the cell in the basal state (Figure 4A, third row), while AQP3 (green) appropriately localized to the basolateral surface [23]. Furthermore, upon dDAVP stimulation, AQP2-F204V failed to translocate to the cell surface (Figure 4A, bottom row). To confirm these findings, the staining was repeated in kidneys taken from two further mice for each class, wild-type or mutant, with or without dDAVP treatment, with identical results.

**Figure 4:**
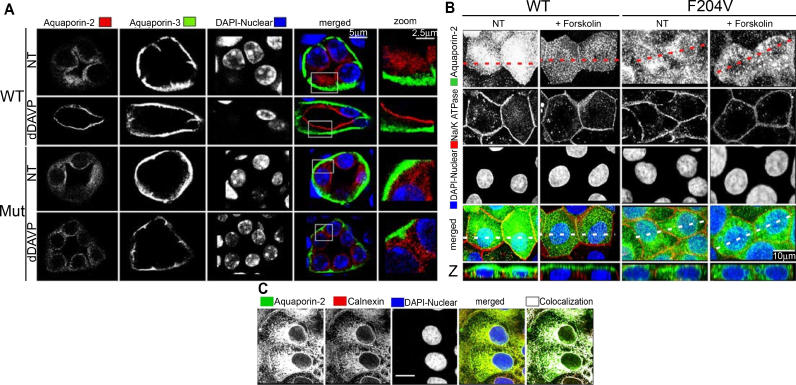
AQP2 Subcellular Localization and Translocation in Mouse Kidney Collecting Ducts and MDCK Cell Lines (A) Immunohistochemistry on collecting ducts in kidney sections from an AQP2-F204V mutant (Mut) mouse and an age-sex matched wild-type (WT) littermate. Mice were injected intraperitoneally with PBS (NT) or dDAVP before sacrificing and fixation of the kidneys. Kidneys sections were immunostained for AQP2 (red) and the basolateral marker AQP3 (green). The images were merged and an area of the cytoplasm was magnified (zoom). Note that mutant AQP2 is not properly localized to the subapical compartment, nor does it respond to dDAVP. (B) MDCK cell lines, stably transfected with constructs encoding mouse WT or AQP2-F204V, were treated with and without 150 μM forskolin for 90 min, after which cells were fixed, permeabilized, and subjected to immunocytochemistry. AQP2 is shown in green, and the basolateral marker Na^+^/K^+^-ATPase is shown in red, alongside the nuclear stain DAPI. The z-profile images were reconstructed from multiple z-sections, along the dotted line. Mutant AQP2 fails to localize to the cell surface upon forskolin stimulation. Rather, the perinuclear staining is consistent with an ER localization of mutant AQP2. (C) The MDCK cell line expressing AQP2-F204V was grown on fibronectin-coated coverslips until tight junctions formed, at which point the cells were treated with 150 μM forskolin for 90 min. Cells were fixed, permeabilized, and sequentially immunoblotted for AQP2 (green) and calnexin (red), an ER marker. The merged image shows that AQP2-F204V colocalizes with the endoplasmic reticulum marker. Scale bar refers to 10 μm.

To investigate the mechanism of defective translocation of AQP2-F204V, we turned to transfection of MDCK cells. Stable cell lines expressing mouse wild-type AQP2 and AQP2-F204V were established. Immunoblots of protein extracts from stable cell lines showed that MDCK cells recapitulate the glycosylation defect seen in mutant mice (unpublished data). The wild-type protein was again present in three different forms. Cells expressing AQP2-F204V lacked the 35–45 kDa form and were enriched in the core-glycosylated 31 kDa form.

In transfected, unstimulated MDCK cells, wild-type AQP2 (stained green) appeared in a punctate pattern distributed throughout the subapical region (Figure 4B, left column photomicrographs), consistent with vesicular compartmentalization. AQP2-F204V, on the other hand, appeared in a punctate but perinuclear pattern (Figure 4B, third column). Upon stimulation with forskolin, a cAMP-dependent protein kinase activator, wild-type AQP2 translocated to the apical surface of polarized MDCK cells (Figure 4B, second column). Along the z-axis, the perinuclear distribution of AQP2-F204V was clearly seen, and this distribution is not altered by forskolin (Figure 4B, bottom row, two right columns). The perinuclear distribution of AQP2-F204V is consistent with an ER compartmentalization. To test the idea that AQP2-F204V localizes to the ER, we co-stained cells transfected with *Aqp2^F204V^* (cDNA) for AQP2 and an ER marker, calnexin (Figure 4C). Colocalization of calnexin with AQP2 was investigated directly, and it was found that 80% of all AQP2-F204V protein colocalized with calnexin. The remaining 20% appeared at the periphery of the ER, representing AQP2-F204V that had potentially progressed beyond the ER. This “ER escape” was consistent with the small proportion of mature, complex glycosylated, AQP2-F204V in mutant kidneys (see Figure 3A).

Animals heterozygous for the *Aqp2^F204V^* mutation were not affected in their urine production or urine osmolality (see Figure 1C and 1D). It has also been shown that a recessive NDI allele, AQP2-R187C, does not interact with wild-type protein in oocytes [24], nor does it homo-oligomerize in MDCK cells [22]. Therefore, kidneys from heterozygous animals were examined for evidence of two populations of AQP2 protein. Surprisingly, immunohistochemical staining of kidney collecting ducts from *Aqp2^F204V/+^* mice revealed a pattern remarkably similar to wild type (Figure 5A). AQP2 translocated completely to the apical cell surface upon dDAVP stimulation. This wild-type staining pattern may simply reflect the fact that decreasing the amount of mutant protein by half makes it undetectable by immunocytochemistry. Alternatively, the presence of wild-type protein may alter the localization of the mutant protein. Indeed, Hendriks et al. proposed a “piggy-back” mechanism to explain the transport of nonglycosylated subunits of AQP2 to the cell surface by glycosylated subunits [22]. It has also been shown that wild-type AQP2 protein can rescue a translocation-defective mutant protein, AQP2-P262L, when the two are coexpressed in MDCK cells [25].

**Figure 5:**
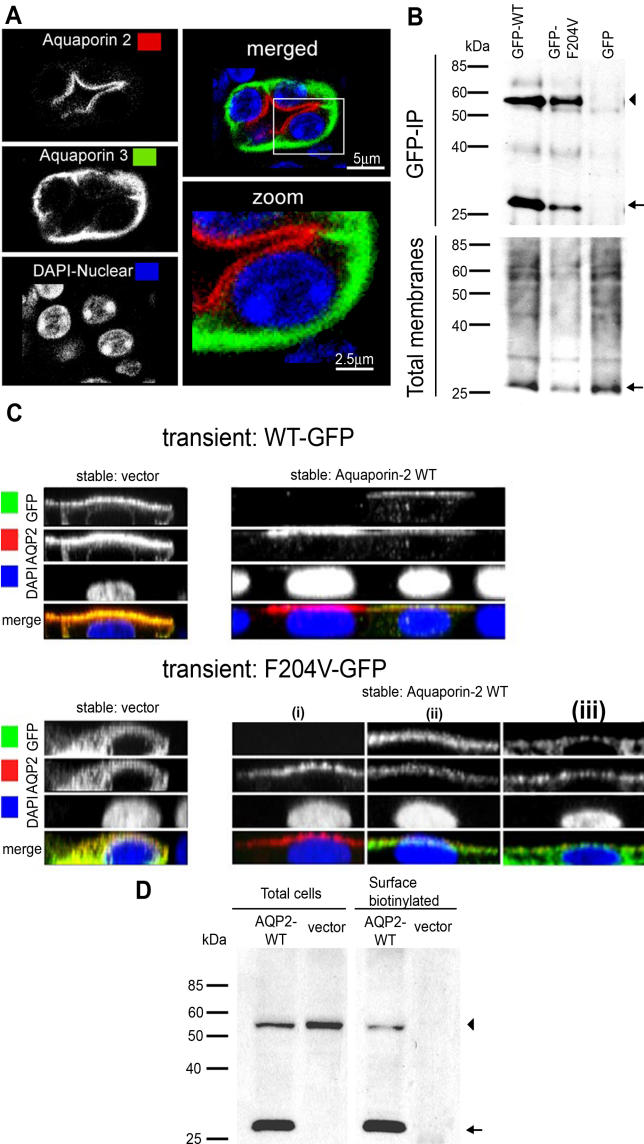
AQP2-F204V Rescue in Heterozygous Mouse Collecting Ducts and in Cotransfected MDCK Cells (A) In heterozygous animals, AQP2 localizes and responds to dDAVP normally. Immunohistochemistry was carried out on kidney sections from an *Aqp2^F204V/+^* mouse, after injection with dDAVP. Kidney sections were sequentially immunostained for AQP2 (red) and the basolateral marker AQP3 (green). (B) Mutant and wild-type AQP2 physically interact. MDCK cells stably expressing wild-type AQP2 were transiently transfected with GFP tagged wild-type AQP2, AQP2-F204V, or GFP alone. Solubilized membranes were immunoprecipitated with a GFP antibody. Total membranes and immunoprecipitates (GFP-IP) were Western blotted using an antibody against AQP2 (arrow) or AQP2-GFP fusions (arrowhead). (C) Wild-type AQP2 rescues the localization defect of mutant AQP2. GFP fusions of either wild-type AQP2 (WT-GFP, top photomicrographs) or F204V AQP2 (F204V-GFP, bottom photomicrographs) were expressed in polarized MDCK stable cell lines expressing vector alone (vector, left photomicrographs) or AQP2-WT (right photomicrographs). Cells were stimulated with forskolin, processed for immunocytochemistry, and used to generate z-sectional images. (D) Mutant AQP2 is present at the cell surface in cells coexpressing wild-type AQP2 (AQP2-WT). GFP fused to AQP2-F204V was expressed in MDCK cells expressing wild-type AQP2 or vector alone. Cells were stimulated with forskolin, and cell surface biotinylated proteins were precipitated then analyzed for the presence of wild-type AQP2 (arrow) and AQP2-F204V (arrowhead) by Western blot.

In the collecting ducts from *Aqp2^F204V/+^* mice, the wild type may rescue the mutant protein as suggested by the subcellular distribution of AQP2 protein. To test this idea, we first looked for an interaction between mutant and wild-type proteins in transfected MDCK cells (Figure 5B). MDCK cells stably expressing wild-type AQP2 were transiently transfected with GFP expression constructs encoding GFP-tagged wild-type AQP2, AQP2-F204V, or GFP alone. Antibodies against GFP coimmunoprecipitated wild-type AQP2 when AQP2-GFP or AQP2-F204V-GFP was transiently transfected, but not when GFP by itself was transiently transfected into MDCK cells stably expressing wild-type AQP2 (Figure 5B, upper blots). Western blot of total membranes showed that wild-type AQP2 is equivalently expressed in all three cases (Figure 5B, lower blots).

If wild-type and mutant proteins are indeed interacting in the cell, is this interaction sufficient to rescue the localization of mutant protein? To answer this question, we used MDCK cells stably transfected with wild-type AQP2 expressing vector or with empty vector. On top of these, we transiently transfected AQP2-GFP or AQP2-F204V-GFP expression constructs. AQP2-GFP localized to the apical surface upon forskolin stimulation whether it was transiently transfected into vector-only cells (Figure 5C, upper left images) or into wild-type AQP2 cells (Figure 5C, upper right). AQP2-F204V-GFP, when expressed by transient transfection into vector only cells, showed a diffuse cytoplasmic distribution pattern (Figure 5C, lower left). When expressed in wild-type AQP2 cells, however, AQP2-F204V-GFP localized to the apical surface to varying degrees (Figure 5C, lower right images [i–iii]). The lower right images of Figure 5C shows three cells from a single transfection. The first is a nontransfected cell that shows the localization of the stably expressing wild-type AQP2, which is apical upon forskolin stimulation. The next two show expression of both the stable wild-type AQP2 and the transient AQP2-F204V-GFP. In cell (ii), localization of wild-type AQP2 was indistinguishable from AQP2-F204V-GFP; both were apical upon forskolin stimulation. Although the effect was subtle in cell (iii), AQP2-F204V-GFP was partly localized to the apical surface. Generally, the localization of AQP2-F204V-GFP was clearly more apical when wild-type AQP2 was also expressed.

To confirm these results biochemically, we transfected the same cell lines (wild-type AQP2 or vector) with F204V-GFP, biotinylated surface proteins after forskolin stimulation, and precipitated the biotinylated proteins (Figure 5D). AQP2-F204V-GFP is expressed approximately equally in both cell lines (Figure 5D, total cells), but is biotinylated only when wild-type AQP2 is also expressed (Figure 5D, surface biotinylated). Since only cell surface proteins are accessible to biotin, these results indicate that AQP2-F204V is transported to the cell surface when wild-type AQP2 is present, but not on its own.

## Discussion

*Aqp2^F204/F204V^* mice are viable and grow and reproduce normally. They are, however, severely defective in their ability to concentrate urine, leading to increased urine output and water intake, thus making them the first mouse model of NDI to survive to maturity. In humans, NDI is caused by mutations in *Avpr2* or *Aqp2*. Knockout of the X-linked *Avpr2* gene in mice [15] gave an NDI-like phenotype in male, hemizygous neonates, but the phenotype could not be assessed in adults as the mice died within 1 wk of birth. The adult heterozygous females showed a mild tendency toward increased urinary output and water intake and decreased urine osmolality. Knockout of the mouse *Aqp2* gene has not been reported. A knock-in of a human disease-causing mutation (T126M), however, has been made [18]. These mice have a severe urine-concentrating defect resulting in dehydration and death within 1 wk of birth. Curiously, AQP2-T126M does localize properly in at least a subset of cells. The grossly abnormal collecting duct morphology makes it impossible to pinpoint the molecular defect in these knock-in mice.

The T126M knock-in clearly shows that *Aqp2* is an essential gene [18]. The fact that our mice survive shows either that AQP2-F204V possesses some residual water transporting ability or that there are AVP-independent pathways for water reabsorption. Residual activity of AQP2-F204V is likely, as mutant animals show some small response to dDAVP, although dDAVP-stimulated urine osmolality remains quite low. Immunostaining of kidney shows that AQP2-F204V does not efficiently transport water, because it fails to localize to the apical cell surface after dDAVP treatment. Some residual activity of AQP2 would imply that some small, undetectable portion of the mutant protein is getting to the cell surface. The surface biotinylation experiment (Figure 5D) suggests that no mutant protein gets to the surface, but this does not necessarily reflect the situation in vivo. While this small fraction of protein may not be detectable by immunofluorescence, Western blotting shows that some mutant protein does progress beyond the ER (35–45 kDa species in Figure 3A). Compared to wild-type, mutant protein is enriched in the high-mannose, core-glycosylated form (31 kDa) and deficient in nonglycosylated (29 kDa) and complex glycosylated (35–45 kDa) forms. The presence of a reduced but detectable amount of protein in the 35–45 kDa range indicates that mutant protein is transported out of the ER, but with greatly reduced efficiency. Colocalization of AQP2-F204V with the ER protein calnexin in transfected MDCK cells shows that, while most of the mutant protein is trapped in the ER, some does progress beyond the ER. Diminished response to dDAVP, diminished abundance of mature glycosylated protein in mutant animals, and the transport of a fraction of mutant protein beyond the ER in MDCK cells are all consistent with the notion that AQP2-F204V misfolding is limited and that it may retain some residual water transporting activity. Evidently this residual activity is sufficient for the viability and growth of mutant animals.

Reduced efficiency in exiting the ER may explain why AQP2-F204V is enriched in the 31 kDa high-mannose glycosylated form. The high-mannose core oligosaccharide is added in the ER and is later modified and elaborated in the Golgi apparatus [26]. The increase in the high-mannose glycosylated form of AQP2-F204V may simply reflect its prolonged presence in the ER and exposure to oligosaccharyl transferase.

While improper localization of AQP2 explains the phenotype of homozygous mutant mice, the complete lack of a phenotype in heterozygous mice is more difficult to explain. Physiologically, heterozygous mice have no symptoms (see Figure 1C), and they are indistinguishable from wild type on immunostaining of kidneys (Figure 5A). The presence of 50% of the normal amount of wild-type protein may explain the lack of symptoms, but it cannot explain the lack of any ER-retained mutant protein. Rather, the phenotype of the *Aqp2^F204V/+^* animals suggests that the mutant protein is being rescued by the wild-type protein. Indeed, de Mattia et al. (26) have demonstrated that one recessive allele of *Aqp2,* P262L, does not properly translocate when expressed by itself in MDCK cells, but that in the presence of wild-type protein, it localizes normally. The same mechanism seems to apply in vivo with *Aqp2^F204V/+^* mice. In support of this, AQP2-F204V can interact with wild-type AQP2 (Figure 5B), and when coexpressed with wild-type protein, AQP2-F204V can reach the cell surface (Figure 5C bottom right panel and 5D). Although it has been demonstrated that a recessive allele (encoding AQP2-R187C) of NDI fails to interact with wild-type AQP2 [6], here we show that AQP2-F204V does interact with the wild-type protein, presumably as part of heterotetramers, and represents a rescuable allele, both in vitro and in vivo.

Immunostaining the kidneys of homozygous *Aqp2^F204V/F204V^* mice shows that the mutant-expressing collecting duct cells can not mediate water reabsorption, because it fails to insert into the apical plasma membrane in response to dDAVP. This is this first in vivo proof of a long-standing hypothesis that comes from in vitro studies with recessive *Aqp2* mutations. Transfection into MDCK cells of any of several *Aqp2* mutations corresponding to recessive human alleles shows abnormal subcellular localization [25], [27] and failure to appropriately translocate to the plasma membrane. Thus, misfolding, retention in the ER, and failure to translocate in response to dDAVP were proposed as the mechanism for autosomal recessive NDI. Here we not only prove this hypothesis but also establish a useful model for human NDI. This mouse model of NDI based on an *Aqp2* allele that can be rescued provides the opportunity to test therapies, including gene therapy, that may promote proper subcellular localization.

## Materials and Methods

### Generation of ENU mice and housing.

ENU mutagenized C57BL/6 mice were generated as described [19]. Mice were maintained by backcrossing affected animals to C57BL/6 and housed in the Genomics Institute of the Novartis Research Foundation Specific Pathogen Free animal facility (La Jolla, California, United States). All procedures were approved by the Genomics Institute of the Novartis Research Foundation Institutional Animal Care and Use Committee.

### Constructs.

The complete coding sequence of mouse AQP2 from an IMAGE clone was digested from the pCMV⋅SPORT6 plasmid with EcoRI and NotI and ligated into pcDNA3.1 (Invitrogen, Carlsbad, California, United States). The F204V mutation was introduced by site-directed mutagenesis (Stratagene, La Jolla, California, United States), using the sense oligonucleotide 5′-GATGATCACTGGGTCGTCTGGATCGGACCCC-3′, and antisense oligonucleotide 5′-GGGGTCCGATCCAGACGACCCAGTGATCATC-3′. To generate GFP fusions of AQP2, the pCMV⋅SPORT6 AQP2 construct was used in a PCR reaction with the primers Sp6 and 5′-GACTGGATCCCGGCCTTGCTGCCGCGCGGCAG-3′ to remove the stop codon of AQP2. The product was digested with KpnI and BamHI and ligated into pEGFP-N2 (BD Biosciences, San Diego, California, United States). The F204V mutation was introduced using the same mutagenic oligonucleotides.

### Cell culture and generation of stable cell lines.

MDCK cells (CCL-34; ATCC, Manassas, Virginia, United States) were cultured in DMEM (Sigma-Aldrich, St. Louis, Missouri, United States) supplemented with 10% FBS (Sigma-Aldrich), 100 U/ml of penicillin, and 100 μg/ml of streptomycin at 37 °C in 5% CO_2_. To generate stable MDCK cell lines, cells were transfected using Lipofectamine 2000 (Invitrogen) and the pcDNA3.1 expression constructs (containing wild-type AQP2, AQP2-F204V, or no insert) and selected with 900 μg/ml G418 (Sigma-Aldrich). Individual colonies were expanded 14 d later. For the duration of these experiments, the antibiotic was continually added to the media. Transient GFP transfections were carried out in subconfluent stable cells lines also using Lipofectamine 2000.

### Sequencing of *Aqp2* and genotyping of mice.

All exons of *Aqp2* were amplified from mouse genomic DNA and sequenced. For genotyping, exon 4 was amplified using the primers 5′-TCAGAACTTGCCCACTAGCC-3′ and 5′-TGTAGAGGAGGGAACCGATG-3′.

### Urine measurements.

Total urine output was measured by separately housing adult mice in Nalgene Metabolic Cages (Minimitter, Bend, Oregon, United States) for 2–3 d and collecting urine every 24 h period. Urine osmolalities were determined using an Osmometer (Osmette 5004; Precision Systems, Natick, Massachusetts, United States). Urine concentrating experiments were carried out by intraperitoneal injection of dDAVP (0.4 μg/kg). Mice were injected twice with dDAVP, once at time 0 and again at 30 min. Urine was collected at the start of the experiment and 30 min after the second injection.

### Kidney membrane preparation.

Whole mouse kidneys were homogenized in 10 mM Tris (pH 7.4), 350 mM sucrose, and 5 mM EDTA containing protease inhibitors (Sigma-Aldrich, #P-8340) in a Potter-Elvehjem homogenizer. The homogenate was centrifuged at 2,000 *g* for 10 min and the supernatant was subjected to ultracentrifugation at 100,000 *g* for 1 h at 4 °C. Pelleted membranes were resuspended in the same buffer, and protein concentration was determined by Bradford assay.

### Immunoblotting.

Kidney membrane fractions (60 μg) were resolved on a 12% SDS-polyacrylamide gel and transferred to a nitrocellulose membrane. Membranes were blocked in 5% nonfat milk in Tris-buffered saline with 0.05% Tween 20 (TBST), followed by an overnight incubation (at 4 °C) with AQP2 polyclonal antibody (Santa Cruz Biotechnology, Santa Cruz, California, United States; #sc-9882). Membranes were washed in TBST then incubated with HRP-conjugated donkey anti-goat antibody. Membranes were washed further in TBST and bands were visualized using ECL reagent (Amersham Biosciences, Little Chalfont, United Kingdom).

### Endoglycosidase digestion.

Kidney membranes (60 μg) were incubated in 50 mM sodium phosphate (pH 5.5), 0.1% SDS, and 50 mM β-mercaptoethanol, heated to 100 °C for 5 min, then cooled. Endoglycosidase H (0.01 units; Sigma-Aldrich) was added and incubated at 37 °C for 2 h. The reaction was stopped by boiling the samples in Laemmli buffer. Total reactants were immunoblotted as described above.

### Coimmunoprecipitation and biotinylation in MDCK cells.

MDCK cells stably expressing wild-type AQP2 (grown on 10-cm plates) were transfected with pEGFP-wild-type AQP2, pEGFP-AQP2-F204V, or vector alone. The cells were homogenized in 10 mM Tris (pH 7.4), 1 mM EDTA, and 250 mM sucrose 40 h later. The clarified supernatant was centrifuged at 200,000 *g* for 30 min. Pelleted membranes were resuspended in the same buffer but containing 4% sodium deoxycholate and incubated at 37 °C for 1 h. From the dissolved membranes, a 30 μl sample was removed and used as the total membrane fraction. The remaining membranes were diluted with 600 μl of the homogenization buffer, and incubated with 1 μl of GFP antisera (Invitrogen, #46–0092) and protein A/G sepharose. Following overnight incubation, the precipitated proteins were washed in RIPA buffer and finally boiled in 50 μl of Laemmli buffer. Half of the total membrane and the IP fractions were processed for immunoblotting.

Cell surface biotinylation was performed in a similar manner. However, pEGFP-AQP2-F204V, was transfected into MDCK cells stably expressing wild-type AQP2 and cells made stable with vector alone. Twenty-four hours post-transfection, cells were stimulated with forskolin, trypsinized, resuspended in 1 ml of PBS (2.5 × 10^6^ cells/ml), and incubated with 0.5 mg of NHS-PEO_4_-biotin (Pierce Biotechnology) for 30 min at room temperature. Cells were washed once in 10 mM Tris (pH 8) and three times in PBS, after which membranes were purified and solubilized as described above. Solubilized membranes were incubated with 20 μl of immobilized streptavidin (Pierce Biotechnology) for 2 h at 4 °C. Finally the precipitated proteins were washed in RIPA buffer and boiled in 50 μl of Laemmli buffer. Total cells and the biotinylated precipitates were immunoblotting using an antibody to AQP2.

### Kidney immunohistochemistry.

Whole mouse kidneys were fixed in 10% phosphate-buffered formalin for 24 h. Kidneys were embedded in paraffin, and 5-μm sections were prepared. Following antigen retrieval using 10 mM sodium citrate (pH 8) for 10 min at 98 °C, sections were sequentially probed, first for AQP3 and then for AQP2. Sections were incubated in 5% donkey serum and then in goat anti-AQP3 antibody (1:100; Santa Cruz Biotechnology; #sc-9885). Slides were washed with PBS and incubated with AlexaFluor 488-conjugated donkey anti-goat antibody (Molecular Probes, Eugene, Oregon, United States). The slides were subsequently blocked in 5% chicken serum, incubated with a rabbit anti-AQP2 antibody (1:250; USB, Cleveland, Ohio, United States; #A3000–06), which was detected with a AlexaFluor 594-conjugated chicken anti-rabbit antibody (1:500; Molecular Probes). The sections were stained with DAPI and mounted in Vectashield (Vector Labs, Burlingame, California, United States).

### Immunocytochemistry on MDCK cells.

MDCK stable cell lines expressing vector alone, wild-type AQP2, or AQP2-F204V (and in some cases transiently expressing a GFP construct) were grown on fibronectin-coated coverslips until tight junctions formed. Cells were treated with or without 150 μM forskolin for 90 min, and fixed in methanol at −20 °C. Subsequently, cells were washed and permeabilized in 0.2% Triton X-100 for 5 min, and sequentially probed for AQP2 and organelle markers for either the PM or the ER. AQP2 was detected using goat anti-AQP2 (1:100; Santa Cruz Biotechnology; #sc-9882) and a 1:200 dilution of AlexaFluor 488-conjugated donkey anti-goat secondary antibody. The PM and ER were probed using mouse anti-Na^+^/K^+^-ATPase (Upstate, Waltham, Massachusetts, United States) or rabbit anti-calnexin (Stressgen Biotechnology, Victoria, British Columbia, Canada) antibodies and the secondary antibodies, Cy3-conjugated goat anti-mouse (1:200; Jackson ImmunoResearch, West Grove, Pennsylvania, United States) or AlexaFluor 594-conjugated chicken anti-rabbit (1:200) respectively. Cells were washed in PBS, counterstained with DAPI, and mounted in Vectashield. In experiments in which GFP fusions were used, AQP2 was probed using the antibody combination used for kidney immunohistochemistry in order to detect the AQP2 at 594 nm, to distinguish between the GFP fusion proteins.

### Confocal microscopy.

Optical z-section images were collected on a BioRad (Hercules, California, United States) Rainbow Radiance 2100 Laser Scanning Confocal Microscope. Image stacks were flattened, or sectioned along the z-axis, then further processed using BioRad Laser Sharp 2000 software and Image J software (v. 1.32; National Institutes of Health). Colocalization was performed using the overlay coefficient of Image J software.

## Supporting Information

### Accession Numbers

The GenBank (http://www.ncbi.nlm.nih.gov/) accession number of *Aqp2* is NM_009699. The IMAGE (http://image.llnl.gov) accession number of AQP2 is 4222942.

## Abbreviations

AQP[number]: aquaporin-[number]
AVPR2: AVP type 2 receptor
AVP: arginine vasopressin
dDAVP: 1-deamino-8-D-arginine vasopressin
ER: endoplasmic reticulum
MDCK: Madin-Darby canine kidney
NDI: nephrogenic diabetes insipidus
PM: plasma membrane
